# Injectable Thermo-Responsive Peptide Hydrogels and Its Enzyme Triggered Dynamic Self-Assembly

**DOI:** 10.3390/polym16091221

**Published:** 2024-04-26

**Authors:** Bowen Yin, Ruoxue Wang, Yu Guo, Liuxuan Li, Xiuli Hu

**Affiliations:** Institute of Polymer Science and Engineering, School of Chemical Engineering, Hebei University of Technology, Tianjin 300130, China; yinbw1014@163.com (B.Y.); w13513354867@163.com (R.W.); guoyu200406@163.com (Y.G.); yugali929@163.com (L.L.)

**Keywords:** polypeptides, enzyme responsive, thermo-sensitive, injectable hydrogel, phosphotyrosine

## Abstract

Endogenous stimuli-responsive injectable hydrogels hold significant promise for practical applications due to their spatio-temporal controllable drug delivery. Herein, we report a facile strategy to construct a series of in situ formation polypeptide hydrogels with thermal responsiveness and enzyme-triggered dynamic self-assembly. The thermo-responsive hydrogels are from the diblock random copolymer mPEG-b-P(Glu-co-Tyr). The L-glutamic acid (Glu) segments with different γ-alkyl groups, including methyl, ethyl, and n-butyl, offer specific secondary structure, facilitating the formation of hydrogel. The L-tyrosine (Tyr) residues not only provide hydrogen-bond interactions and thus adjust the sol–gel transition temperatures, but also endow polypeptide enzyme-responsive properties. The PTyr segments could be phosphorylated, and the phosphotyrosine copolymers were amphiphilies, which could readily self-assemble into spherical aggregates and transform into sheet-like structures upon dephosphorylation by alkaline phosphatase (ALP). P(MGlu-co-Tyr/P) and P(MGlu-co-Tyr) copolymers showed good compatibility with both MC3T3-E1 and Hela cells, with cell viability above 80% at concentrations up to 1000 μg/mL. The prepared injectable polypeptide hydrogel and its enzyme-triggered self-assemblies show particular potential for biomedical applications.

## 1. Introduction

Over the past few decades, injectable hydrogels have attracted great interest and are being leveraged for drug delivery, sensors, scaffolds, and actuators [1,2,3,4,5]. The unique injectability confers the feasibility of injection of an aqueous solution of polymers into the body, followed by in situ gelation driven by specific physiologically relevant endogenous or exogenous stimuli. In contrast to systemic delivery, locally delivered therapies offer more advantages in terms of minimal adverse effects, minimal invasive administration procedures, and spatio-temporal controlled drug delivery and release [6,7,8,9]. Among the various stimuli, such as temperature, redox signals, pH, enzymes, and glucose, that have been employed for sol-to-gel phase transitions, temperature is of particular interest for its advantages in practical applications [10,11,12,13,14,15,16,17]. Thus, thermosensitive hydrogels that have a mild sol-to-gel over-behavior and convert to a gel state at body temperatures are highly desirable.

Inspired by the self-assembly and diverse functions of nature proteins and peptides, synthetic polypeptides that have analogical compositions and structures similar to nature proteins are potential building blocks for the formation of supramolecular hydrogels due to their unique secondary structures such as random coil, α-helix and β-sheet and their responsiveness to physiological signals or external stimuli [11,18]. The inherent compatibility, tunable biodegradability, controllable secondary structure, versatility, and scalable synthetic strategy of polypeptides also contribute to their wide applications as depots for local drug delivery, antibacterial materials, and scaffolds [7,11,19,20,21,22,23]. The sol-to-gel transition temperature is highly influenced by the secondary structures and composites of synthetic polypeptides [24,25]. Therefore, how to tailor the sol-to-gel transition temperature and the mechanical properties of hydrogels via regulation of secondary structures is proposed [26,27]. Polypeptides based on Glu have been extensively investigated, and polyglutamate derivatives have a propensity to adopt α-helical rigid conformation because of the intramolecular hydrogen bond [28,29,30]. Serine and tyrosine prefer to form β-sheets due to their intermolecular hydrogen bonding interactions [31,32,33]. The α-helical domain commonly exists in protein networks involving mechanical signals. The β-sheet is mainly found in amyloids, silk, and titin [34,35]. For example, spider silks comprise 10–15% domains of antiparallel β-sheet crystals embedded in a hydrophilic protein matrix. This nanoconfinement structure endows spider silks with high stiffness and toughness [36]. Inspired by spider silks, random copolymers based on different amino acids may provide a facile strategy to develop injectable hydrogels with diverse structures and mechanical properties. Moreover, tyrosine can be modified by phosphorylation, and controlling the conformation of proteins by phosphorylation/dephosphorylation can further modulate the biological activity of proteins [37]. It has been reported in the literature that phosphorylation/dephosphorylation efficiently regulates the folding of amphiphilic short peptides to form a fiber network conformation [38]. We extend this concept to the polymers studied in this paper.

Herein, we report a facile strategy to construct a series of in situ injectable reversible polypeptide hydrogels with thermal responsiveness for local injection by ring opening polymerization (ROP) of γ-alkyl-L-glutamic acid N-carboxyanhydride (Glu-NCA) and L-tyrosine N-carboxyanhydride (Tyr-NCA) using mPEG as an initiator to obtain block copolymer mPEG-b-P(Glu-co-Tyr) at a low degree of polymerization (DP ≤ 18), providing satisfactory dispersion or aqueous solubility. The PGlu segments present both α-helix and β-sheet secondary structures, facilitating the formation of hydrogel. PTyr segments with phenolic groups provide polar side chains and additional intermolecular hydrogen bonds with pendant carbonyls of PGlu segments or water molecules, which promotes the self-assembly of polypeptides. It is worth mentioning that the PTyr could also be phosphorylated, and the corresponding phosphorylated polypeptide could respond to alkaline phosphatase (ALP) and conduct dynamic self-assembly.

## 2. Materials and Methods

### 2.1. Materials and Characterization

Methoxy polyethylene glycol amino (mPEG_45_-NH_2_, Mn = 2000) was purchased from Pengsheng Biological Company (Shanghai, China) and used without further purification. _L_-glutamic acid (L-GA) and _L_-tyrosine (L-Tyr) were purchased from Macklin Biochemical Company (Shanghai, China). ALP was obtained from BiDe Pharmaceutical Technology Company (Shanghai, China). Ultra-dry dimethylformamide (DMF), ultra-dry triethylamine, and ultra-dry phosphorous oxychloride were purchased from Anhui Zesheng Technology Company (Anhui, China). Phosphate buffer (PBS) pH 7.4 was purchased from Servicebio (Wuhan, China). All other reagents and solvents were purchased from Komeo Chemical Reagent (Tianjin, China), and all other reagents were used directly without further purification. HeLa (a human cervical cancer cell line) and MC3T3-E1 (mouse embryonic osteoblast cell line) were purchased from the American Type Culture Collection (ATCC, Rockville, MD, USA). HeLa cells were cultured in Dulbecco’s Modified Eagle Medium (DMEM, Gibco, Grand Island, NY, USA) containing 10% fetal bovine serum (FBS), and MC3T3-E1 cells were cultured in α-MEM culture medium containing 10% FBS.

Nuclear magnetic resonance hydrogen (^1^H NMR) (Bruker, AVANCE400, Karlsruhe, Germany, GRE) spectrometer and Fourier transform infrared (FTIR) spectroscopy (Bruker, Vetor-22, Karlsruhe, Germany, GRE) were used to characterize the chemical structure of compounds. Dynamic light scattering (DLS) (Malvern, Zetasizer S90, Malvern, UK) was used to determine the zeta potential of the product. Scanning electron microscopy (SEM) (FEI, Nova Nano SEM 450, Portland, OR, USA) was used observed the morphology of polymers. The secondary structure determination of copolymers was characterized by circular dichroism (CD) (Applied Photophysics, Chirascan CD, Shanghai, China) spectroscopy. The dynamic mechanical properties of hydrogels were characterized by rheology tests, which were performed using a rotational rheometer (Anton Paar, MCR-92, Graz, Austria). The 450 nm absorbance of the surviving cells was measured by the Bio-Rad 680 enzyme labeler (Agilent, BioTek Epoch 2, Santa Clara, CA, USA). The optical photographs of cell morphology were recorded by a trinocular biological microscope (Weiyi Optical Instrument, WYS-41XD, Tianjin, China).

### 2.2. Synthesis of PEG-b-P(MGlu-co-Tyr) Copolymer

The synthesis of PEG-b-P(MGlu-co-Tyr) (abbreviated as P(MGlu-co-Tyr)) block copolymers was prepared by ROP of different proportions of Tyr NCA and MGlu NCA using mPEG_45_-NH_2_ as the macroinitiator. Briefly, Tyr NCA (0.93 g, 4.5 mmol), MGlu NCA (1.83 g, 9.75 mmol), and mPEG_45_-NH_2_ (1.50 g, 0.75 mmol) were dissolved in anhydrous N, N-dimethylformamide (DMF) under a nitrogen atmosphere. The reaction was stirred for 72 h at 35 °C and then precipitated into diethyl ether. The obtained P(MGlu-co-Tyr) was filtered and dried at room temperature in vacuo. The yield was 71%. ^1^H NMR (400 MHz, TFA-d) δ 7.16–6.95 (m, 6H, -C_6_**H**_4_-), 6.93–6.77 (m, 6H, -C_6_**H**_4_-), 4.78 (dd, 17H, -COC**H**NH-), 3.90 (d, 180H, -**PEG**), 3.80 (d, 46H, -C**H**_3_), 2.65–2.54 (m, 30H, -C**H**_2_C_6_H_4_-), 2.34–2.24 (m, 17H, -COOC**H**_2_-), 2.14 (d, 18H, -NHCHC**H**_2_-).

### 2.3. Synthesis of PEG-b-P(MGlu-co-Tyr/P)

P(MGlu-co-Tyr) (50 mg) was dissolved in anhydrous N-Methyl Pyrrolidone. Anhydrous triethylamine (200 μL) and phosphoryl chloride (200 μL) were then added. The reaction was stirred overnight, and then a sodium bicarbonate solution was added. PEG-b-(MGlu-co-Tyr/P) (abbreviated as P(MGlu-co-Tyr/P)) was obtained by dialysis (molecular weight cutoff: 3500 Da) against deionized water for 2 days and then lyophilized. (Yield: 63%). ^1^H NMR (400 MHz, TFA-d) δ 7.31 (s, 21H, -**H**_2_PO_4_), 4.85 (s, 9H, -COC**H**NH-), 4.43 (s, 5H, -COC**H**NH-), 3.97 (s, 180H, -**PEG**), 3.81 (d, 38H, -C**H**_3_), 3.38 (p, 26H, -NHCHCH_2_C**H**_2_-), 3.09 (s, 14H, -C**H**_2_C_6_H_4_-), 2.59 (d, 33H, -NHCHC**H**_2_-).

### 2.4. Gel Phase Diagram and Gel Time Measurement

The sol-to-gel transition behavior of the copolymers was determined by the vial inversion method. A certain concentration of polymer solution was prepared with PBS (pH 7.4) and added to a vial. Then, the polymer solution was stirred in an ice bath for more than 36 h to form a homogeneous system. Finally, the temperature was increased by 1 °C every ten minutes by the gradient temperature increase method, and the maximum temperature was set at 60 °C. The gel temperature was recorded through the vial inversion method by observing that there was no flow within half a minute (*N* = 3). To determine the gelation time, vials containing the polymer solutions at 0 °C were immersed directly into a 37 °C water bath. The vial was taken out every 5 s and inverted to observe whether the solution flowed. The gelation time was recorded when stable hydrogels formed. Each data point from the above test represented the average and standard deviation of three measurements.

### 2.5. Rheology Test

The dynamic mechanical properties of hydrogels were characterized by rheology tests, which were performed using a rotational rheometer. The PBS 7.4 aqueous polymer solution was placed between parallel plates of 25 mm diameter with a gap of 1.0 mm. The edge of the sample was covered with silicone oil to prevent water evaporation. The data were collected under 1% strain and 1.0 rad/s. Temperature test range of 10–70 °C for PGlu hydrogels and 10–60 °C for P(MGlu-co-Tyr) hydrogels, and the heating rate was 0.5 °C/min. In addition, to further investigate the stability of hydrogels, the variation of hydrogel modulus with angular frequency at physiological temperature was tested.

### 2.6. In Vitro Cytotoxicity Analysis

The cytotoxicity was detected by the Cell Counting Kit-8 (CCK8) assay. The copolymers were configured with pH 7.4 PBS as 31.25 μg/mL, 62.5 μg/mL, 125 μg/mL, 250 μg/mL, 500 μg/mL, and 1000 μg/mL solutions and irradiated with UV light for 2 h. MC3T3-E1 or HeLa cells were inoculated into 96-well plates (8000 cells/well), and 180 μL of DMEM or α-MEM was added to each well, and incubated overnight at 37 °C in an atmosphere of 5% CO_2_. Then the medium was removed, and a new 180 μL of medium was added to each well. Polymer solution (20 μL) was added to each well, and PBS was used as the control group. After 48 h, the CCK8 assay was performed by measuring the absorbance of the surviving cells with the BioTek Epoch 2 enzyme labeler (450 nm). Cell viability (%) was evaluated by standardizing the absorbance of the samples to the absorbance of control wells. The measurements were repeated three times. According to previously reported literature [39], the formula for calculating cell viability is expressed as follows:

$$\mathrm{Cell}\;\mathrm{viability}\left(\%\right)=\left(\frac{A_{\mathrm{sample}}-A_{\mathrm{blank}}}{A_{\mathrm{control}}-A_{\mathrm{blank}}}\right)\times 100$$

where A_sample_ refers to the absorption value of the sample wells, A_blank_ is the absorption value with the addition of an equal volume of medium, and A_control_ refers to the absorption value of the sample wells with a polymer concentration of zero.

### 2.7. ALP Triggered Dynamic Self-Assembly

P(MGlu-co-Tyr/P) (20 mg) was incubated with ALP (5U/mL) in Tris HCl buffer (pH 8.8) under 37 °C for 24 h. Afterwards, the mixture was dialyzed (molecular weight cutoff: 3500 Da) against deionized water for 2 days to remove residual ALP. The micromorphology, zeta potential, and secondary structure before and after ALP-mediated assembly were observed by SEM, DLS, and circular dichroism spectroscopy (CD). The details of the experiment are as follows:

A tiny amount of P(MGlu-co-Tyr/P) aqueous solution and P(MGlu-co-Tyr/P) aqueous solution incubated with ALP and dialyzed to remove the residual ALP were taken on clean silicon wafers, dried, and used SEM to observe the microscopic morphology. 1 mL of P(MGlu-co-Tyr/P) aqueous solution and P(MGlu-co-Tyr/P) aqueous solution incubated with ALP and dialyzed to remove the residual ALP were placed in a Malvern zeta potential cuvette, respectively, and the zeta potentials were tested by DLS. P(MGlu-co-Tyr/P) and P(MGlu-co-Tyr/P) treated with ALP incubation and dialyzed to remove excess ALP were diluted to a concentration of 0.5 mg/mL with deionized water, mixed homogeneously, and tested by circular dichroism at 20 °C, respectively.

## 3. Results

### 3.1. Synthesis and Characterization of Copolymers

**Scheme 1:**
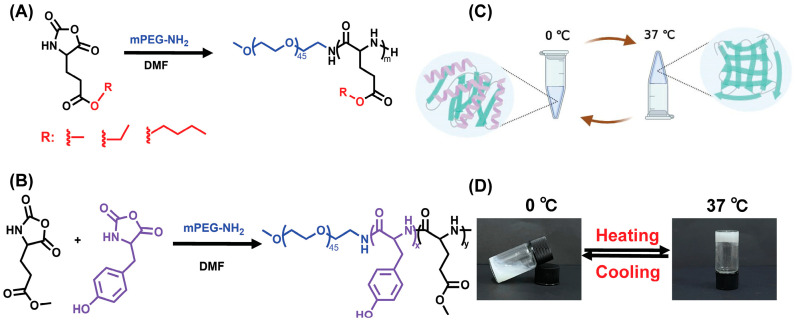
Schematic illustration of polypeptide synthesis and hydrogel formation. (**A**) Synthetic routes of poly(ethylene glycol)-block-poly(γ-alkyl-L-glutamate). (**B**) Synthetic routes of P(MGlu-co-Tyr) copolymers. (**C**) Gel mechanism of reversible peptide hydrogels. (**D**) Photographs of reversible phase transitions of P(MGlu-co-Tyr) copolymer solution at 8.0 wt % at 0 °C and 37 °C, respectively.

The block copolymer poly(ethylene glycol)-*block*-poly(γ-alkyl-L-glutamate) (mPEG-b-PGlu) (abbreviated as PGlu) with different functional groups including methyl, ethyl, and n-butyl was synthesized by ROP of the corresponding γ-methyl-L-glutamate N-carboxyanhydride (MGlu NCA), γ-ethyl-L-glutamate N-carboxyanhydride (EGlu NCA), and γ-butyl-L-glutamate N-carboxyanhydride (BGlu NCA) using methoxy mPEG_45_-NH_2_ as a macroinitiator (Scheme 1A). The obtained copolymers were abbreviated as PMGlu, PEGlu, and PBGlu, respectively. L-tyrosine N-carboxyanhydride (Tyr-NCA) monomer could also be introduced to prepare copolymer P(MGlu-co-Tyr) (Scheme 1B). The DP of each segment was controlled by adjusting the feed molar ratio of the NCA monomer during polymerization (Table S1). The copolymer solution could form gels with an increase in temperature, and the sol-to-gel transition was reversible (Scheme 1C). Scheme 1D presents photographs of the reversible sol-to-gel transition of P(MGlu-co-Tyr). Next, the composition and structure of the obtained copolymer were characterized by ^1^H NMR spectra and FTIR spectra.

**Figure 1:**
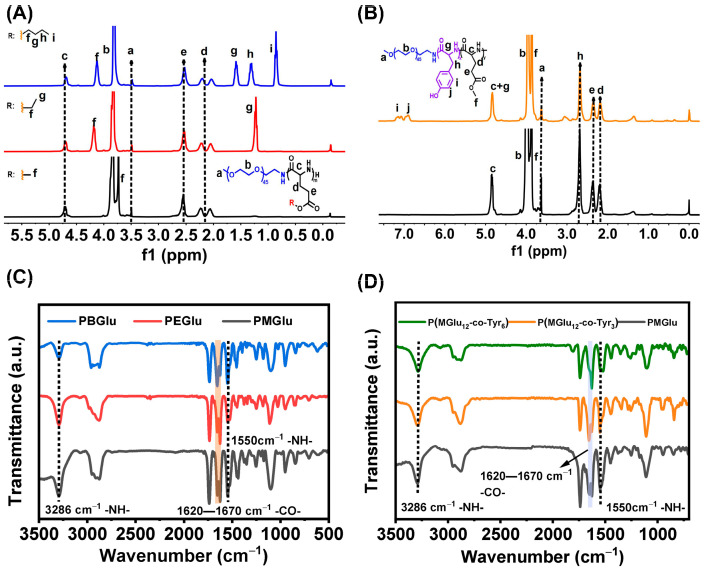
Characterization of PGlu derivatives and P(MGlu-co-Tyr) copolymers. (**A**) ^1^H NMR of spectra of PMGlu, PEGlu and PBGlu in TFA-d. (**B**) ^1^H NMR of spectra of PMGlu and P(MGlu-co-Tyr) in TFA-d. (**C**) FTIR spectra of PMGlu, PEGlu and PBGlu in the solid state. (**D**) FTIR spectra of PMGlu and P(MGlu-co-Tyr) in the solid state.

^1^H NMR spectra confirmed the successful synthesis of the four kinds of NCA monomers and their corresponding PGlu derivatives (including PMGlu, PEGlu, and PBGlu) (Figure S1 and Figure 1A). The Tyr segment was successfully introduced by copolymerization of MGlu NCA and Tyr-NCA monomers, as shown in Figure 1B. The composition and molecular weight of the peptide blocks were calculated by the integrals of ^1^H NMR spectra at 3.70−3.80 ppm (−CH_3_ of MGlu), 1.20−1.25 ppm (−CH_2_CH_3_ of EGlu), 0.82−0.88 ppm (−CH_2_CH_2_CH_2_CH_3_ of BGlu), 6.76−7.16 ppm (phenyl group of Tyr), 3.80−3.95 ppm (−CH_2_CH_2_O− of PEG) (Figure 1A,B). The DP of copolymers was determined by ^1^H NMR, as shown in Table S1. Figure 1C and Figure S2 displayed FTIR spectra of the polypeptides PMGlu, PEGlu, and PBGlu and the corresponding monomers MGlu NCA, EGlu NCA, and BGlu NCA at room temperature, respectively. The successful synthesis of polymers was evidenced by the appearance of new absorbance at 1652, 1627 cm^−1^, representing the stretching vibration of -C=O- on the main chain, peaks at 1550 cm^−1^ for the in-plane bending vibration of -NH-, and the disappearance of the characteristic peaks at 1745–1880 cm^−1^ from the C=O groups of NCA rings. The ^1^H NMR and FTIR spectroscopic analyses indicated the successful synthesis of PMGlu, PEGlu, PBGlu, and P(MGlu-co-Tyr).

### 3.2. Gel Phase Diagram

**Figure 2:**
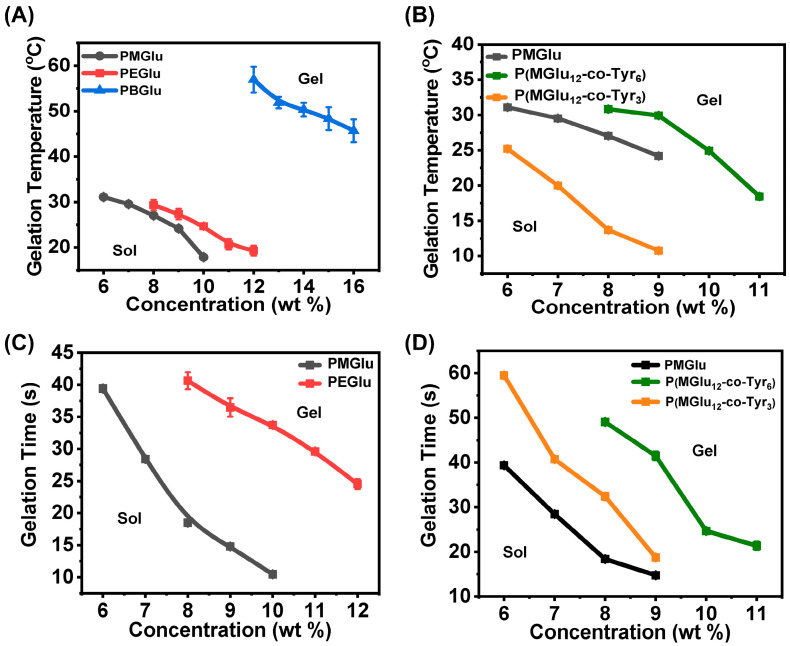
(**A**) Sol-to-gel phase diagrams of PMGlu, PEGlu, and PBGlu in PBS 7.4 solutions. (**B**) Sol-to-gel phase diagrams of PMGlu, P(MGlu_12_-co-Tyr_3_) and P(MGlu_12_-co-Tyr_6_) copolymers in PBS 7.4 solutions. (**C**) Gelation time of PMGlu and PEGlu at different concentrations. (**D**) Gelation time of PMGlu, P(MGlu_12_-co-Tyr_3_), and P(MGlu_12_-co-Tyr_6_) at different concentrations.

The thermo-responsive gelation behaviors of PGlu derivatives and L-tyrosine copolymers P(MGlu-co-Tyr) were investigated with the test tube inverting method by the flow (sol) and no flow (gel) standard. The phase diagrams of block copolymers with different side chains (PMGlu, PEGlu, and PBGlu) and different compositions (PMGlu-co-Tyr) are shown in Figure 2A,B. As shown in Figure 2A, all these polymers underwent a thermo-responsive sol-to-gel transition at a concentration range of 6.0−16.0 wt % within a temperature range of 10 to 60 °C. The sol-to-gel transition temperature depends on the polymer concentrations and decreases with increasing polymer concentrations. The alkyl functional groups have great influence on the thermo-responsive gelation behaviors.

As shown in Figure 2A, PMGlu and PEGlu had similar sol-to-gel transition temperatures; however, PBGlu showed much higher transition temperatures. For example, at the concentration of 10 wt%, the critical gelation temperatures (CGT) were 17 °C and 24 °C for PMGlu and PEGlu_,_ respectively, and PBGlu could not form gels at this concentration. Based on its excellent gelation ability, PMGlu was selected for copolymerization with Tyr-NCA to obtain P(MGlu_12_-co-Tyr_3_) and P(MGlu_12_-co-Tyr_6_) with different compositions. As shown in Figure 2B, the introduction of a small amount of tyrosine segments (P(MGlu_12_-co-Tyr_3_)) helps to enhance the gelation ability compared with PMGlu, thereby reducing the CGT. However, the sol-to-gel CGT increases as the composition of tyrosine increases. Considering the same DP of PEG segments for all polymers, the difference in sol-to-gel transition temperature was mainly attributed to the changes in the secondary structure and intramolecular hydrogen bonding caused by copolymerization. In general, sol-to-gel phase transition temperatures are correlated with the side-chain groups, hydrogen-bond interactions, and secondary structures [13]. The gelation time of different copolymer solutions was tested by increasing the temperature directly from 0 °C to 37 °C (Figure 2C,D). The gelation time of all five copolymers exhibited similar trends to the phase diagrams, which means that the hydrogelation time changes with the polymer concentration and composition. All these polymers could form hydrogels within 1 min, and all the gels are injectable, ensuring their operability for practical applications.

### 3.3. Gel Mechanism and Secondary Structure

**Figure 3:**
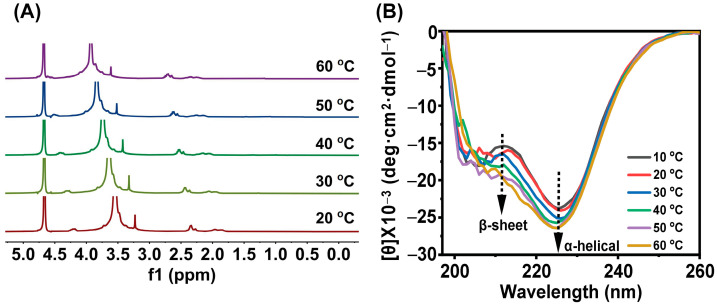
(**A**) ^1^H NMR spectra of PMGlu aqueous solutions (D_2_O) as a function of temperature. (**B**) CD spectra of PMGlu aqueous solution (0.5 mg/mL) as a function of temperature.

In order to investigate the mechanism of the thermo-responsive transition, temperature-dependent ^1^H NMR and CD spectroscopy of PMGlu were recorded to explore the secondary structure of peptide hydrogels. According to the variable-temperature ^1^H NMR spectra in Figure 3A, the PEG characteristic peaks at 3.50 ppm shifted downfield as the temperature increased from 20 to 60 °C, suggesting the gradual dehydration and restricted motion of the PEG segment with increasing temperature [24]. According to the literature [40], the CD spectra of typical α-helical structure have the lowest peaks at 208 nm and 222 nm, and the characteristic peaks of typical β-sheet structure have the lowest peak near 216 nm and a positive peak near 196 nm. When α-helix and β-sheet secondary structures coexist, the peak shape and characteristic peaks change slightly [41]. The CD spectra of PMGlu aqueous solution in Figure 3B showed a negative band at 226 nm and a negative shoulder at 212 nm, corresponding to both α-helical and β-sheet. As the temperature increased from 10 to 60 °C, β-sheet and α-helical conformations became stronger. The β-sheet and α-helix conformations arise from intermolecular and intramolecular hydrogen binding interactions, respectively. The sol-to-gel transition mechanism was mainly attributed to the dehydration of hydrophilic PEG segments and the increment of hierarchically ordered structure, including β-sheet and α-helix, at higher temperatures. The properties of side chain groups and the composition of copolymers both have significant impacts on the secondary structure of polypeptides [24,28].

**Figure 4:**
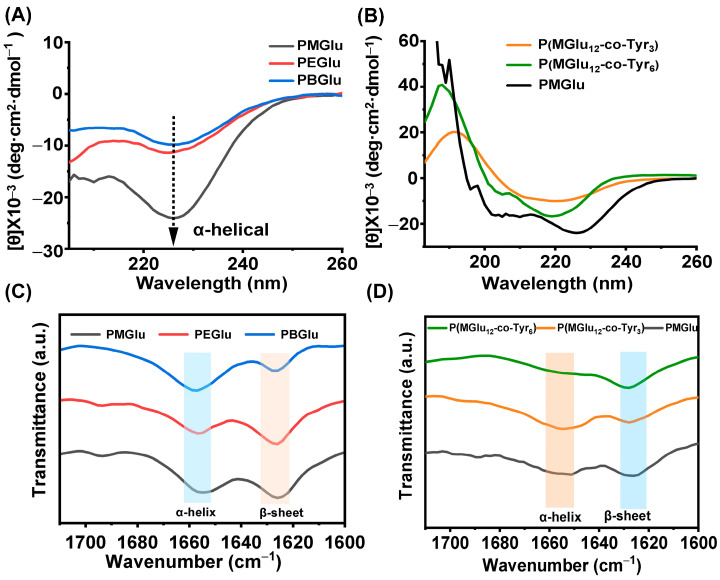
(**A**) CD spectra of PMGlu, PEGlu, and PBGlu in aqueous solution (0.5 mg/mL) at 20 °C. (**B**) CD spectra of PMGlu, P(MGlu_12_-co-Tyr_3_), and P(MGlu_12_-co-Tyr_6_) copolymers in aqueous solution (0.5 mg/mL) at 20 °C. (**C**) FTIR spectra of PMGlu, PEGlu, and PBGlu copolymers in the solid state. (**D**) FTIR spectra of PMGlu, P(MGlu_12_-co-Tyr_3_), and P(MGlu_12_-co-Tyr_6_) copolymers in the solid state.

CD was used for the structure analysis of polypeptides. The CD pattern showed negative valleys at 226 nm, confirming α-helical conformation for all three PGlu polypeptides, as shown in Figure 4A [28]. As the substituent changes from methyl to butyl, the degree of helicity decreases. With the introduction of Tyr segments, the conformation of P(MGlu-co-Tyr) gradually changed from α-helical to β-sheet, as the characteristic minima at 226 nm transformed to 216 nm in the CD spectra (Figure 4B). Tyr has been reported to have intermolecular hydrogen bond interaction and a propensity to form β-sheet conformation [42,43]. So the difference between sol-to-gel CGT in Figure 2B could explain that Tyr segments provide an increment of hydrogen bonds and β-sheet conformation, which is beneficial for gel formation. Conversely, the secondary structures of PMGlu block will be destroyed with the increase in the content of Tyr, which is not conducive to the formation of hydrogel, and P(MGlu_12_-co-Tyr_6_) presents a higher phase transition temperature compared with PMGlu. FTIR spectroscopy was carried out in the solid state to further investigate the secondary structure of the polymers. Figure 4C shows the scale-expanded FTIR spectra of the amide stretching regions of PMGlu, PEGlu, and PBGlu, and the amide stretching frequencies were mainly split into three bands: 1626 cm^−1^ and 1653–1660 cm^−1^, corresponding to β-sheet and α-helical secondary structures, respectively. FTIR spectra further confirmed that the copolymerization with Tyr segments changed the secondary structure of polypeptides. With the increase in Tyr contents, β-sheet conformation increased (Figure 4D). All these results confirm that the length and sequence of amino acid monomers influence the secondary structures of polypeptides, which render the supramolecular self-assembly of polypeptides into a three-dimensional network structure.

### 3.4. Rheology Test

**Figure 5:**
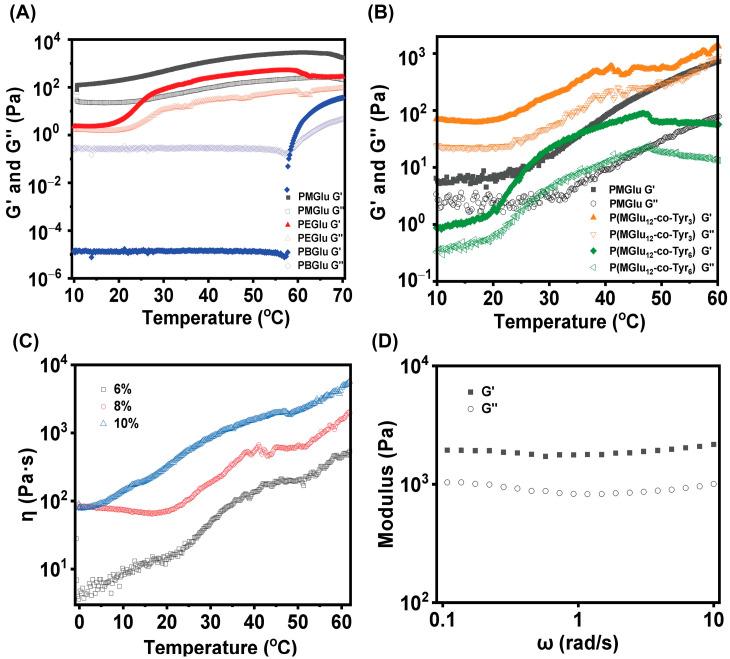
(**A**) G′ and G″ of PMGlu, PEGlu, and PBGlu in 9.0 wt% PBS aqueous solutions as a function of temperature. (**B**) G′ and G″ of PMGlu, P(MGlu_12_-co-Tyr_3_), and P(MGlu_12_-co-Tyr_6_) in 8.0 wt% PBS aqueous solutions as a function of temperature. (**C**) Viscosity of P(MGlu_12_-co-Tyr_3_) as a function of temperature. (**D**) Frequency scan of P(MGlu_12_-co-Tyr_3_) at 9% concentration.

The dynamic mechanical properties of polymers with different side chain groups and compositions were evaluated by the rheological tests. First, the storage modulus (G′) and loss modulus (G′′) of PMGlu, PEGlu, and PBGlu (9.0 wt % aqueous solution) were recorded with the increase in temperature. As shown in Figure 5A, PBGlu was present as sol with low G′ when the temperature was between 10 and 60 °C. When the temperature was increased to nearly 60 °C, the G′ increased sharply. The crossing point of G′ over G′′ represents the sol–gel transition temperature, which was much higher than that of PMGlu and PEGlu. The block copolymer PMGlu showed the highest G′ in the initial state and the strongest gelation ability compared with PEGlu and PBGlu, which coincided well with the sol–gel transition temperature and gelation time results. Notably, the G′ of PMGlu hydrogel (136 Pa at 10 °C) is almost 6 orders of magnitude higher than that of PBGlu hydrogel (1.29 × 10^−5^ Pa at 10 °C), indicating the important impact of pendant groups on hydrogel performance. With the introduction of the Tyr segment, G′ was generally higher than G″ for the three copolymers P(MGlu_12_-co-Tyr_3_), PMGlu, and P(MGlu_12_-co-Tyr_6_) hydrogels, indicating the elastic nature. Importantly, the G′ values of these three polymers are in the order of P(MGlu_12_-co-Tyr_3_) > PMGlu > P(MGlu_12_-co-Tyr_6_), implying that the viscoelastic properties of polypeptide hydrogels could be tailored by the specific composition and concentration of precursor hydrogels (Figure 5B). PGlu and P (MGlu-co-Tyr) hydrogels are physically crosslinked hydrogels formed through hydrophobic interactions [5,44], so that their G′ is generally low and their mechanical properties perform poorly [45]. However, they can be constructed with gelatin, cellulose, etc., to build a composite hydrogel and improve their mechanical properties [45,46]. In addition, we also investigated the variation of viscosity with polymer concentrations. As shown in Figure 5C and Figure S3, the viscosity increases with the increase in P(MGlu-co-Tyr) and PMGlu polymer concentration. G′ and G′′ of the hydrogels maintain constant in the scanning range of 0.1–10 angular frequencies, indicating good stability of peptide hydrogels (Figure 5D). The injectability of hydrogel was shown in Video S1. The microstructure of the hydrogels was characterized by SEM (Figure S4). The hydrogels exhibited interconnected, three-dimensional mesh-like porous structures, and these interconnected porous structures may facilitate the transport and release of drugs from the hydrogels.

### 3.5. ALP Triggered Dynamic Self-Assembly

**Figure 6:**
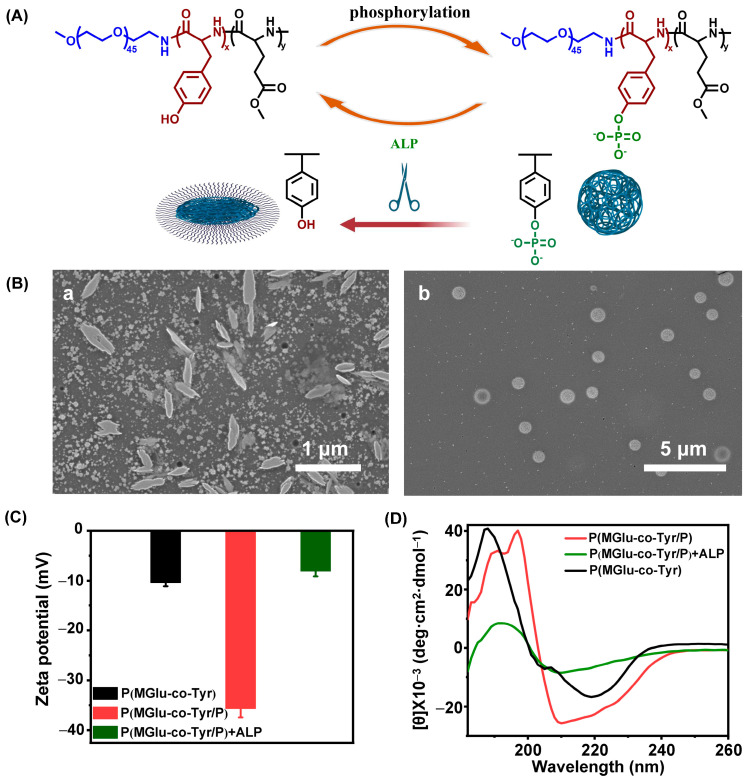
(**A**) The chemical structure of P(MGlu-co-Tyr) and P(MGlu-co-Tyr/P) and schematic diagram of self-assembly properties. (**B**) SEM images of (**a**) P(MGlu-co-Tyr/P) and P(MGlu-co-Tyr/P) incubation with ALP. Scale bars = 1 μm (**a**) and 5 μm (**b**). (**C**) Zeta potential of P(MGlu-co-Tyr), P(MGlu-co-Tyr/P) and P(MGlu-co-Tyr/P) incubation with ALP. (**D**) CD spectra of P(MGlu-co-Tyr), P(MGlu-co-Tyr/P), and incubation with ALP.

Polypeptide phosphorylation commonly occurs at tyrosine, threonine, and serine residues. Phosphorylation of tyrosine is particularly interesting due to its critical role in the pathway for intracellular signaling [15,47,48]. The construction of phosphotyrosine polypeptides is promising for various biological applications. Phosphoric functionalized polypeptide (P(MGlu-co-Tyr/P)) was obtained by esterification of phenol with phosphoryl chloride [49]. ^1^H NMR spectra revealed that the protons of the phenol ring shifted downfield after reaction, proving that tyrosine was successfully phosphorylated in P(MGlu-co-Tyr/P) (Figure S5). The zeta potentials of different copolymers were characterized by DLS. As shown in Figure 6C, the zeta potential of P(MGlu-co-Tyr/P) was significantly lower than that of P(MGlu-co-Tyr) owing to the modification with phosphate groups. The cytotoxicity of P(MGlu-co-Tyr/P) and P(MGlu-co-Tyr) was further evaluated by incubation with MC3T3-E1 and HeLa cell lines for 48 h with various concentrations of P(MGlu-co-Tyr/P) and P(MGlu-co-Tyr). The relative viabilities of MC3T3-E1 and HeLa cells treated with P(MGlu-co-Tyr/P) and P(MGlu-co-Tyr) were measured by CCK8 assay and normalized to the viability of cells treated with medium. Figure S6 depicts that P(MGlu-co-Tyr/P) and P(MGlu-co-Tyr) have no obvious toxicity to both MC3T3-E1 and HeLa cells at concentrations up to 1000 μg/mL, implying that P(MGlu-co-Tyr/P) and P(MGlu-co-Tyr) are safe for biological applications.

Phosphatase, especially ALP, is an active enzyme for hydrolyzing phosphate ester groups from various substrates and has been widely explored for selectively inhibiting cancer cells or drug delivery [50,51,52,53]. Figure 6A is a schematic representation of P(MGlu-co-Tyr/P) self-assembly via ALP. To evaluate the sensitivity of P(MGlu-co-Tyr/P) towards ALP, P(MGlu-co-Tyr/P) solution in PBS (pH 7.4) was incubated with ALP (5 U) for 24 h, and the morphology of the polymer solution was observed using SEM. As shown in Figure 6B, P(MGlu-co-Tyr/P) incubated with ALP for 24 h displayed sheet-like structures, while P(MGlu-co-Tyr/P) exhibited uniform spherical morphology in PBS 7.4. The spherical morphology may be attributed to the excellent solubility of phosphoric acid groups in aqueous solutions and the enhancement of intermolecular forces. While the sheet-like structures derive from the enzymatic dephosphorylation of P(MGlu-co-Tyr/P), inducing the aggregation of P(MGlu-co-Tyr). As shown in Figure 6C, after incubation with ALP, the zeta potential of P(MGlu-co-Tyr/P) increased substantially and was nearly the same as P(MGlu-co-Tyr). In addition, CD was used to record the secondary structure of P(MGlu-co-Tyr) before and after phosphorylation (Figure 6D). The β-sheet conformation was significantly enhanced, as evidenced by the appearance of a new sharp peak at 197 nm and the enhancement of the negative peak at 209 nm after phosphorylation. The secondary structure transition of P(MGlu-co-Tyr/P) could be attributed to the negative charge interactions of the pendant phosphoric acid groups. After incubation with ALP, the β-sheet conformation was significantly weakened.

## 4. Conclusions

In summary, the present work explored different strategies for the design of thermo-responsive polypeptide hydrogels based on poly(glutamic acid) with different pendant groups and random copolypeptides of glutamic acid and tyrosine. The obtained copolymers could undergo thermo-responsive sol-to-gel transitions, and the gel behavior can be tailored by adjusting the block length and compositions. The sol-to-gel transition mechanism was mainly attributed to the dehydration of hydrophilic PEG segments and the formation of hierarchically ordered α-helix and β-sheet structures, as evidenced by CD spectroscopy and FTIR spectroscopy. Notably, the random copolypeptides with tyrosine segments could be partially modified by the phosphate group, and the phosphorylation polypeptide could respond to ALP and conduct dynamic assembly. The present work is helpful for understanding and designing synthetic copolypeptide hydrogels, further exploring their potential biomedical applications.

## Data Availability

Data are contained within the article.

## Supplementary Materials

The following supporting information can be downloaded at: https://www.mdpi.com/article/10.3390/polym16091221/s1, Figure S1: ^1^H NMR spectrum of monomers; Figure S2: FTIR spectra of monomers; Figure S3: Viscosity of PMGlu as a function of temperature. Figure S4: SEM of the P(MGlu-co-Tyr) hydrogel. Figure S5: ^1^H NMR spectra of P(MGlu-co-Tyr) and P(MGlu-co-Tyr/P) in TFA-d. Figure S6: In vitro cytotoxicities of P(MGlu-co-Tyr) and P(MGlu-co-Tyr/P) to MC3T3-E1 (a) and HeLa cells (b). (c) The optical image of MC3T3-E1 cells morphology. (scale bar = 100 μm) (d) The optical image of Hela cells morphology. (scale bar = 100 μm). Table S1: Polymers molecular weight characterization by ^1^H NMR.; Video S1: The injectability of P(MGlu-co-Tyr) hydrogel.
