# Supplementary material for: Injectable Thermo-Responsive Peptide Hydrogels and Its Enzyme Triggered Dynamic Self-Assembly
Source: Polymers (Basel). 2024 Apr 26;16(9):1221. doi: 10.3390/polym16091221 (PMC11085460; doi:10.3390/polym16091221)
Supplement: Supplementary file 1 [file polymers-16-01221-s001.zip › polymers-2969993-supplementary.pdf]

Supporting Information

Injectable Thermo-Responsive Peptide Hydrogels and Its Enzyme Triggered Dynamic Self-Assembly

*Bowen Yin,[1] Ruoxue Wang,[1] Yu guo,[1] Liuxuan Li,[1] Xiuli Hu [1,] **

[1] Institute of Polymer Science and Engineering, School of Chemical Engineering, Hebei University of Technology, Tianjin 300130, China;

* Corresponding authors: huxiuli@hebut.edu.cn (X. Hu).

Table of Contents:

1. Experimental section..3

1.1 Synthesis of γ-methyl-L-glutamate...3

1.2 Synthesis of L-tyrosine N-carboxyanhydride (Tyr-NCA). 4

1.3 Synthesis of MGlu NCA, EGlu NCA and BGlu NCA. 4

1.4 Synthesis of mPEG-b-PGlu 5

1.5 Microscopic morphology of hydrogels..6

2. Supplementary Table...7

3. Supplementary Figures 8

4. Supplementary Video...11

5. References...12

1. Experimental section.

1.1 Synthesis of γ-methyl-L-glutamic acid.

Synthesis of γ-methyl-L-glutamic acid was performed according to an adapted procedure from literature [1] L-glutamic acid (10 g) was added to a round-bottomed flask with 20 mL of methanol, and the ingredients were mixed well and cooled to 0°C. Then 4 mL of 98% concentrated sulfuric acid was slowly added. The reaction was continued for 30 minutes and then stirred at 50°C overnight. The resulting colorless viscous liquid was slowly poured into a mixture of triethylamine and ethanol (1:1 (v:v)) to produce a white precipitate, which was centrifuged to give the crude product. The crude product was dissolved in water, recrystallized in a large amount of ethanol for 3 times, filtered and dried under vacuum to obtain γ-methyl-L-glutamic acid (MGlu) (7.5 g, 68% yield). ^{1}H NMR (400 MHz, D_2O) δ 3.78 (t, 1H, -COC**H**NH-), 3.72 (s, 3H, -C**H**$_3$-), 2.62 – 2.54 (m, 2H, -C**H**$_2$-), 2.17 (d, 2H, -C**H**$_2$-). γ-Ethyl-L-Glutamic acid (EGlu) and γ-n-Butyl-L-Glutamic acid (BGlu) was synthesized similarly to MGlu except that methanol was replaced with ethanol and n-butanol, respectively. (EGlu: 63% yield). ^{1}H NMR (400 MHz, D_2O) δ 4.17 (q, 2H, -C**H**$_2$CH$_3$-), 3.78 (t, 1H, -COC**H**NH-), 2.60 – 2.51 (m, 2H, -COC**H**$_2$CH$_2$-), 2.21 – 2.10 (m, 2H, -C**H**$_2$CH$_2$NH$_2$-), 1.26 (t, 3H, -C**H**$_3$-). (BGlu: 61.5% yield). ^{1}H NMR (400 MHz, D_2O) δ 4.15 (t, 2H, -OC**H**$_2$CH$_2$-), 3.77 (t, 1H, -COC**H**NH$_2$-), 2.60 – 2.53 (m,

2H, -COC**H**$_2$-), 2.20 – 2.12 (m, 2H, -C**H**$_2$CHNH$_2$-), 1.68 – 1.58 (m, 2H,-OCH$_2$C**H**$_2$-), 1.43 – 1.31 (m, 2H,-C**H**$_2$CH$_3$-), 0.91 (t, 3H,-C**H**$_3$).

1.2 Synthesis of L-tyrosine N-carboxyanhydride (Tyr-NCA).

L-tyrosine (5 g, 5.5 mmol) and propylene oxide (21.5 mL, 0.31 mol) were added in THF. Then triphosgene (4.5 g, 15 mmol) was added to the reaction solution. After stirring for 6 h at 35 °C, the reaction solution was concentrated, and precipitated in petroleum ether to give crude Tyr-NCA. The crude product following dissolving in ethyl acetate was washed with cold saturated NaCl aqueous solution and cold water sequentially, and dried with anhydrous $MgSO_4$. Then the product was recrystallized three times with THF and hexane. Yield: 78%. ^{1}H NMR (400 MHz, DMSO-d$_6$) δ 9.32 (s, 1H, -O**H**), 9.02 (s, 1H, -N**H**-), 6.96 (d, 2H, -C$_6$**H**$_5$OH), 6.68 (d, 2H, -C$_6$**H**$_5$CH$_2$), 4.70 (t, 1H, -NHC**H**CO-), 2.95-2.85 (m, 2H, -C**H**$_2$CH-).

1.3 Synthesis of MGlu NCA, EGlu NCA and BGlu NCA.

MGlu (5 g, 31.1 mmoL) was added to a round bottom flask with 80 mL of tetrahydrofuran, and were mixed homogeneously, then triphosgene (9.17 g, 31.1 mmoL) was added and reacted at room temperature for 3 h. The reaction solution was concentrated and settled in a large amount of n-hexane for 3 h, and the crude product MGlu NCA was obtained. The crude product was dissolved in ethyl acetate, washed with distilled water for 3 times and dried with anhydrous sodium sulfate for 3 h, filtered, and the filtrate was concentrated and recrystallized in a large amount of hexane. The filtrate was filtered and dried in vacuum to give MGlu NCA as a white

solid (3.68 g, 63.5% yield). ^{1}H NMR (400 MHz, DMSO-d_6) δ 4.49-4.43 (m, 1H, -NHC**H**-), 3.60 (s, 3H, -C**H**$_3$), 2.45 (t, 2H, -C**H**$_2$COO-), 2.07-1.98 (m, 2H, -C**H**$_2$CHNH-). EGlu NCA and BGlu NCA was synthesized similarly to MGlu NCA except that MGlu was replaced with EGlu and BGlu, respectively. (EGlu NCA: 64.5 % yield) ^{1}H NMR (400 MHz, $CDCl_3$) δ 7.08 (s, 1H, -N**H**-), 4.43 (t, 1H,-NHC**H**-), 4.13 (q, 2H, -C**H**$_2$CH$_3$), 2.52 (t, 2H,-C**H**$_2$COO-), 2.23 (dq, 1H, -C**H**$_2$CHNH-), 2.12 (dt, 1H, -C**H**$_2$CHNH-), 1.25 (t, 3H, -C**H**$_3$).BLG NCA yield is 45.3%, ^{1}H NMR (400 MHz, $CDCl_3$) δ 6.65 (s, 1H, -N**H-**), 4.44-4.37 (m, 1H, -NHC**H**-), 4.10 (td, 2H, -OC**H**$_2$CH$_2$-), 2.55 (t, 2H,- C**H**$_2$COO-), 2.34-2.21 (m, 1H,-C**H**$_2$CHNH-), 2.16-2.06 (m, 1H, -C**H**$_2$CHNH-), 1.62 (p, 2H,-CH$_2$C**H**$_2$CH$_2$-), 1.43-1.31 (m, 2H,-C**H**$_2$CH$_3$), 0.93 (td, 3H,-C**H**$_3$).

1.4 Synthesis of mPEG-b-PGlu

The synthesis of mPEG-b-PGlu (abbreviated as PGlu) with different functional groups including methyl, ethyl, and n-butyl was synthesized through ring-opening polymerization (ROP) of γ-alkyl-L-glutamic acid N-carboxyanhydrides using mPEG$_{45}$-NH$_2$ as a macroinitiator. The steps for the preparation of PMGlu are as follows: mPEG$_{45}$-NH$_2$ (500 mg, 0.25 mmol) and MGlu NCA (750 mg, 4 mmol) were dissolved in anhydrous N, N-dimethylformamide (DMF) under a nitrogen atmosphere. The reaction mixture was stirred at 35 °C for 72 h. Then the reaction mixture was precipitated in glacial diethyl ether, filtered and washed twice repeatedly with glacial diethyl ether. The obtained product was dried under vacuum. Meanwhile,

PEGlu, and PBGlu were synthesized through a similar procedure while the MGlu NCA were replaced with EGlu NCA and BGlu NCA, respectively.

PMGlu: ^{1}H NMR (400 MHz, TFA-d) δ 4.70 (dd, 15H, -COC**H**NH-), 3.83 (s, 180H, -**PEG**), 3.74 (d, 46H, -COOC**H**$_3$-), 2.67 – 2.46 (m, 31H, -C**H**$_2$COO-), 2.24 (t, 15H, -NHCHC**H**$_2$-), 2.06 (p, 15H, -NHCHC**H**$_2$-).

PEGlu: ^{1}H NMR (400 MHz, TFA-d) δ 4.70 (dd, 15H, -COC**H**NH-), 4.22 – 4.16 (m, 31H, CH$_3$C**H**$_2$-), 3.82 (d, 180H, -**PEG**), 2.54 (p, 32H, -COOC**H**$_2$-), 2.29 – 2.15 (m, 17H, -NHCHC**H**$_2$-), 2.12 – 1.97 (m, 15H, -NHCHC**H**$_2$-), 1.22 (td, 46H, -CH$_2$C**H**$_3$).

PBGlu: ^{1}H NMR (400 MHz, TFA-d) δ 4.70 (dd, 15H, -COC**H**NH-), 4.13 (qd, 32H, -COOC**H**$_2$-), 3.81 (d, 180H, -**PEG**), 2.53 (q, 33H, -NHCHCH$_2$C**H**$_2$-), 2.21 (d, 16H, -NHCHC**H**$_2$-), 2.03 (d, 15H, -NHCHC**H**$_2$-), 1.58 (q, 32H, -C**H**$_2$CH$_2$CH$_3$), 1.31 (q, 32H, -C**H**$_2$CH$_3$), 0.85 (dd, 46H, -C**H**$_3$).

1.5 Microscopic morphology of hydrogels.

The 8.0 wt % (w/v) solution of P(MGlu-co-Tyr) was configured with pH 7.4 PBS, stirred in an ice bath to form a homogeneous system, and kept at 37 °C for 10 min. After the solution formed a gel, the hydrogel was placed in liquid nitrogen and frozen for several minutes, after which the hydrogel sample was completely dried by a vacuum freeze dryer, and finally the hydrogels were put into liquid nitrogen to continue to freeze for a few minutes to be cut. The sample was dipped in cross-section onto conductive adhesive, sprayed with carbon under vacuum conditions, and placed in a field emission Scanning Electron Microscope (SEM) for cross-section scanning.

2. Supplementary Table

Table S1. Polymers molecular weight characterization by ^{1}H NMR.

Copolymers	Feeding molar ratio of NCA/$mPEG_{45}$-NH_2	DP[a]	Mn[a]（kDa）
PMGlu	16	15	4.2
PEGlu	16	15	4.4
PBGlu	16	15	4.8
P($MGlu_{12}$-co-Tyr_3)	1/13/3	12/3	4.3
P($MGlu_{12}$-co-Tyr_6)	1/13/6	12/6	4.8

[a] Determined by ^{1} H NMR.

3. Supplementary Figures

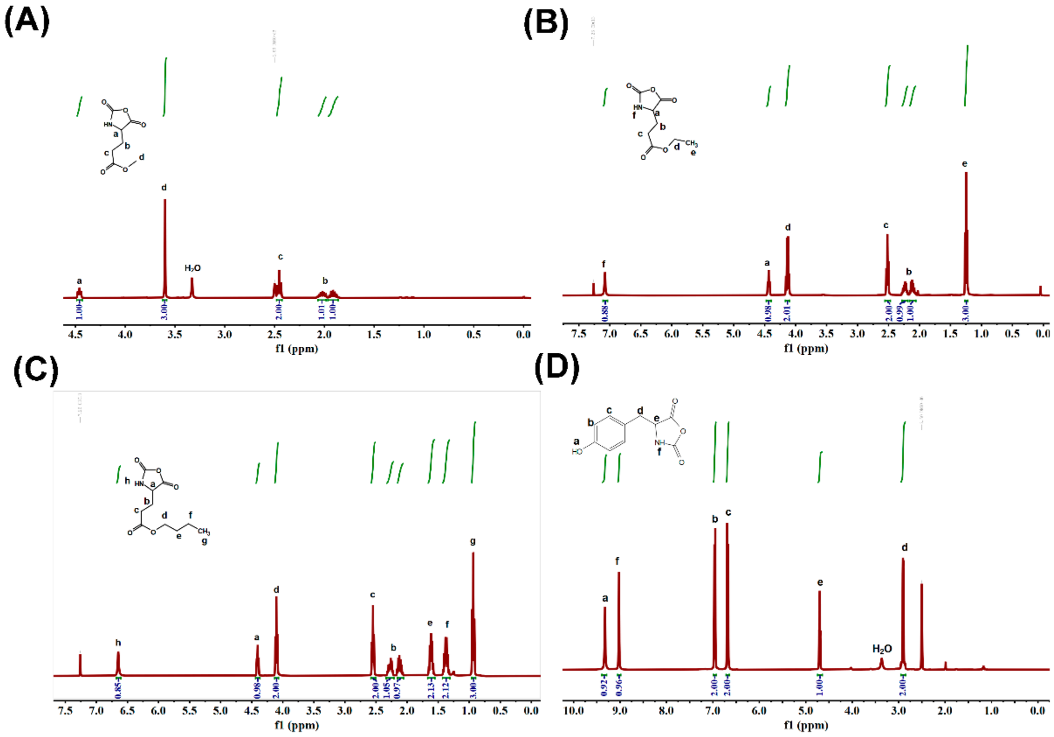

Figure S1. ^{1}H NMR spectrum of (a) MLG NCA (400 MHz, DMSO-d$_6$), (b) ELG NCA (400 MHz, $CDCl_3$), (c) BLG NCA (400 MHz, $CDCl_3$), (d) Tyr-NCA (400 MHz, DMSO-d$_6$).

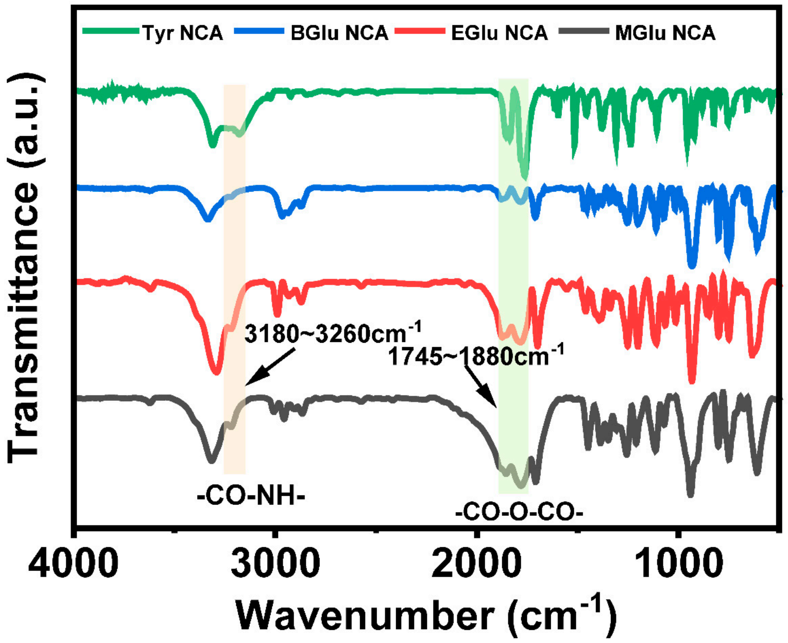

Figure S2. FTIR spectra of Tyr NCA, MGlu NCA, EGlu NCA, and BGlu NCA.

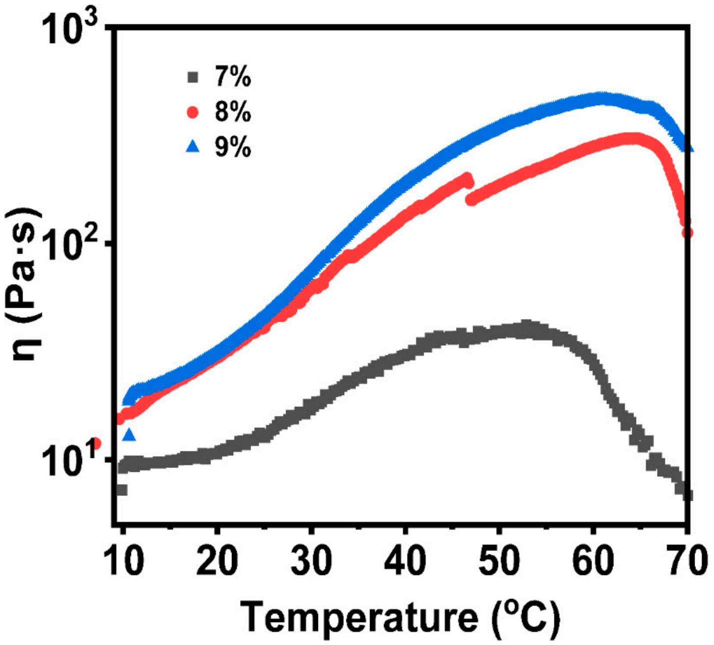

Figure S3. Viscosity of PMGlu as a function of temperature.

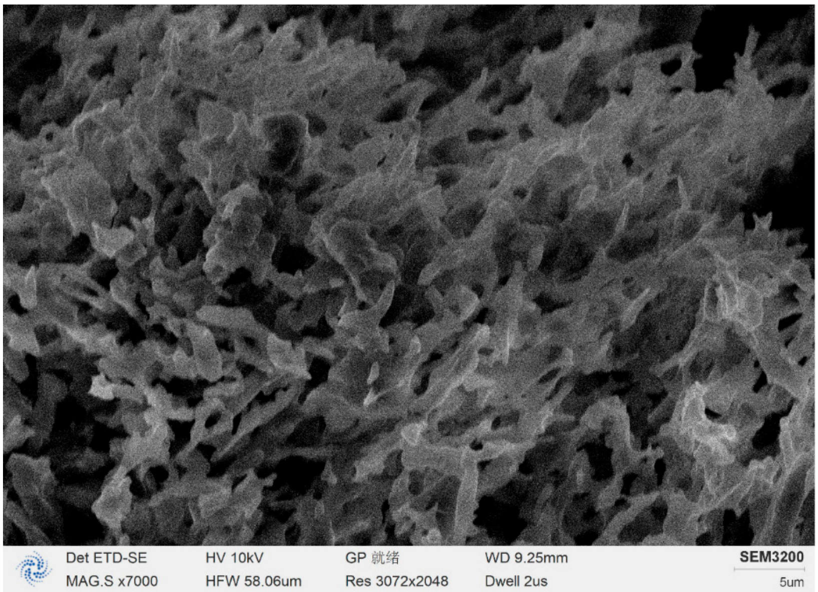

Figure S4. Morphology characterization of the P(MGlu-co-Tyr) hydrogel. (scale bar = 5 μm).

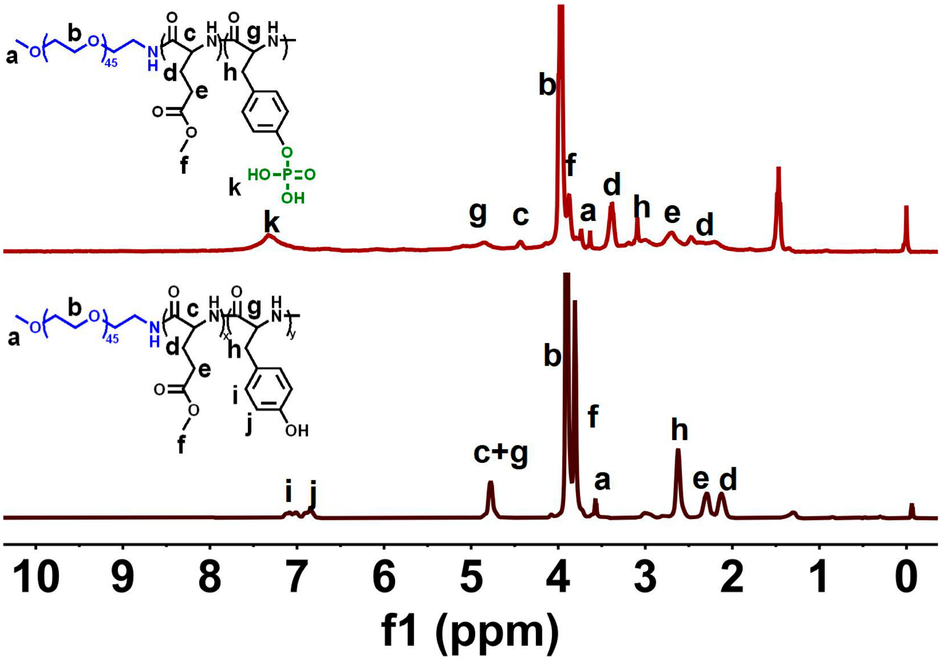

Figure S5. ^{1}H NMR spectra of P(MGlu-co-Tyr) and P(MGlu-co-Tyr/P) in TFA-d.

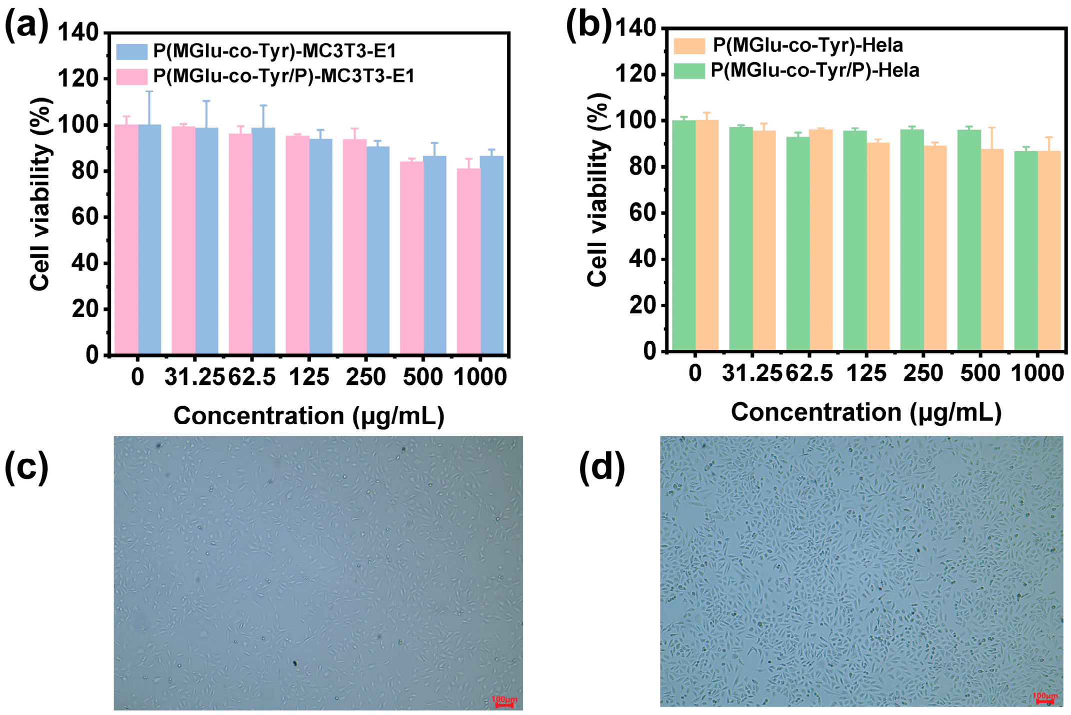

Figure S6. In vitro cytotoxicities of P(MGlu-co-Tyr) and P(MGlu-co-Tyr/P) to MC3T3-E1 (a) and HeLa cells (b). Data are presented as the average ± standard deviation (n=3). (c) The optical image of MC3T3-E1 cells morphology. (scale bar = 100 μm) (d) The optical image of Hela cells morphology. (scale bar = 100 μm).

4. Supplementary Video

Video S1. The injectability of P(MGlu-co-Tyr) hydrogel.

5. References

1. Li, D.; Zhao, D.; He, C. L., Crucial impact of residue chirality on the gelation process and biodegradability of thermoresponsive polypeptide hydrogels. *Biomacromolecules* **2021**, *22,* 3992-4003.
